# 1,2,3,4,5,6,7,8,13,13,14,14-Dodeca­chloro-1,4,4a,4b,5,8,8a,12b-octa­hydro-1,4:5,8-dimethano­triphenyl­ene at 90 K^1^

**DOI:** 10.1107/S1600536812032540

**Published:** 2012-07-25

**Authors:** Brandon W. Jenkins, Frank R. Fronczek, Steven F. Watkins

**Affiliations:** a Department of Chemistry, Louisiana State University, Baton Rouge, LA 70803-1804, USA

## Abstract

The previously reported room-temperature crystal structure [Jaud Baldy, Negrel, Poite & Chanon (1993 ▶). *Z. Kristallogr.* **204**, 289–291] of the title compound, C_20_H_8_Cl_12_, is monoclinic with *Z*′ = 1, whereas the 90 K structure reported herein is triclinic with *Z*′ = 2 and shows a 2% volume contraction. The crystallographically independent unit chosen consists of both enanti­omers (Λ and Δ) of this propeller-like mol­ecule. Both enanti­omers display quasi-twofold symmetry, with average bond-length/bond-angle deviations of 0.0018 (4) Å and 0.41 (2)° for Λ, and 0.0026 (4) Å and 0.50 (2)° for Δ.

## Related literature

For the structure of the room-temperature polymorph, see: Jaud *et al.* (1993 ▶). For the preparation of the compound, see: Lacourcelle *et al.* (1993 ▶). For the Cambridge Structural Database, see: Allen (2002 ▶).
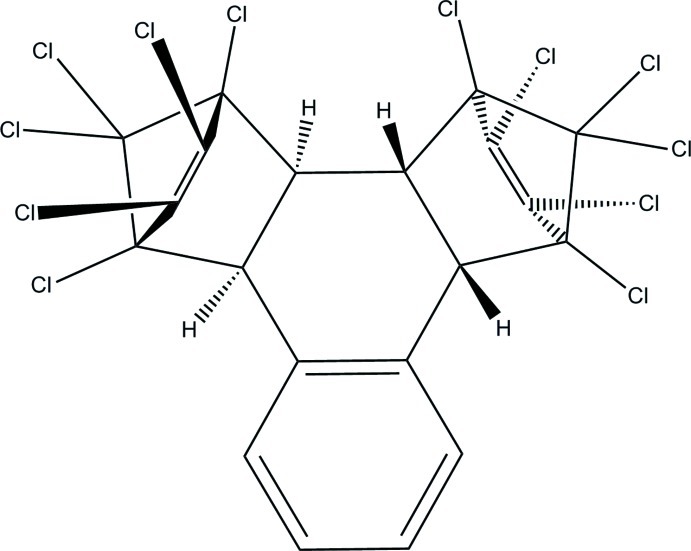


## Experimental

### 

#### Crystal data

C_20_H_8_Cl_12_
*M* *_r_* = 673.66
Triclinic, 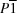

*a* = 9.6434 (1) Å
*b* = 15.4287 (2) Å
*c* = 16.4161 (2) Å
α = 92.1948 (6)°
β = 98.2331 (7)°
γ = 91.3097 (6)°
*V* = 2414.53 (5) Å^3^
*Z* = 4
Mo *K*α radiation
μ = 1.39 mm^−1^
*T* = 90 K
0.40 × 0.35 × 0.15 mm

#### Data collection

Nonius KappaCCD diffractometer
Absorption correction: multi-scan (*SCALEPACK*; Otwinowski & Minor, 1997 ▶) *T*_min_ = 0.607, *T*_max_ = 0.819
111692 measured reflections
30125 independent reflections
24699 reflections with *I* > 2σ(*I*)
*R*_int_ = 0.026

#### Refinement

*R*[*F* ^2^ > 2σ(*F* ^2^)] = 0.032
*wR*(*F* ^2^) = 0.086
*S* = 1.03
30125 reflections
578 parameters
H-atom parameters constrained
Δρ_max_ = 0.87 e Å^−3^
Δρ_min_ = −0.63 e Å^−3^

### 

Data collection: *COLLECT* (Nonius, 2000 ▶); cell refinement: *SCALEPACK* (Otwinowski & Minor, 1997 ▶); data reduction: *DENZO* (Otwinowski & Minor, 1997 ▶) and *SCALEPACK*; program(s) used to solve structure: *SHELXS97* (Sheldrick, 2008 ▶); program(s) used to refine structure: *SHELXL97* (Sheldrick, 2008 ▶); molecular graphics: *ORTEP-3 for Windows* (Farrugia, 1997 ▶); software used to prepare material for publication: *WinGX* (Farrugia, 1999 ▶).

## Supplementary Material

Crystal structure: contains datablock(s) global, I. DOI: 10.1107/S1600536812032540/zj2089sup1.cif

Structure factors: contains datablock(s) I. DOI: 10.1107/S1600536812032540/zj2089Isup2.hkl

Additional supplementary materials:  crystallographic information; 3D view; checkCIF report

## Figures and Tables

**Fig. 1.**
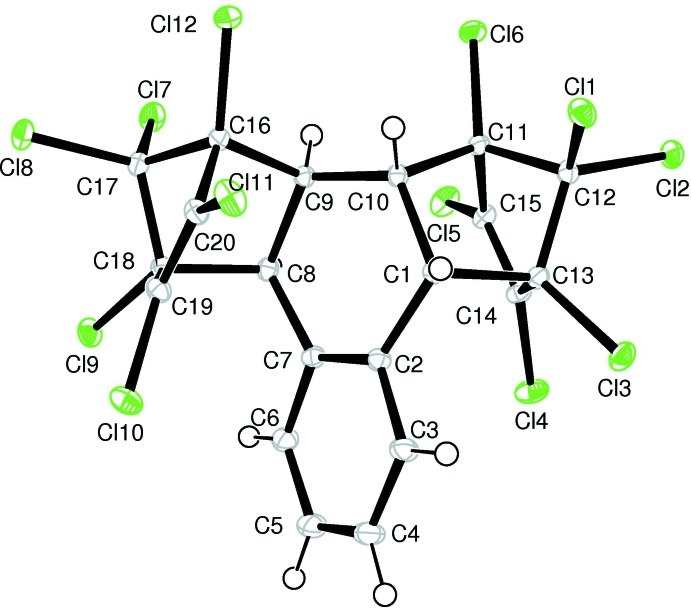
View of Ia (Δ, 50% probability displacement ellipsoids)

**Fig. 2.**
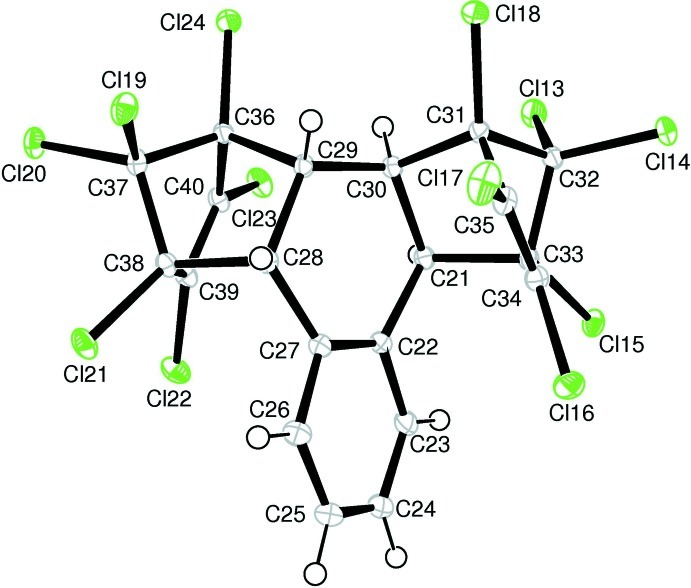
View of Ib (Λ, 50% probability displacement ellipsoids)

## supplementary crystallographic information

### Comment

The crystal structure of the title compound at 298 K is monoclinic, *P*2_1_/*c*, *Z* = 4, a = 9.743 (3), b = 15.523 (3), c = 16.575 (2) Å, β = 100.11 (2)°, in agreement with the published room-temperature structure (Jaud *et al.*, 1993. CCDC refcode YASKOS, Allen, 2002). Upon cooling to 90 K, the material undergoes a phase change to triclinic (*P*1) and a volume contraction of 2%. The two crystallographically independent enantiomers chosen for the asymmetric unit are propeller-like with opposite pitch (Δ, Ia and Λ, Ib). Both display near twofold symmetry, with average off-symmetry bond-length/bond-angle deviations of 0.0018 (4) Å/0.41 (2)° (Ia) and 0.0026 (4) Å/0.50 (2)° (Ib). The bond lengths and bond angles are normal and are essentially as described by Jaud *et al.* (1993).

### Experimental

The compound was prepared by Lee Shui and Mark McLaughlin using the method described by Lacourcelle *et al.* (1993). A sample suitable for diffraction was recrystallized from ethyl acetate.

### Refinement

All H atoms were placed in calculated positions, guided by difference maps, with C—H bond distances 0.95 (aromatic C) and 1.00 (alkyl C) Å, *U*_iso_ = 1.2*U*_eq_, and thereafter refined as riding.

### Crystal data

**Table:**

C_20_H_8_Cl_12_	*Z* = 4
*M**_r_* = 673.66	*F*(000) = 1328
Triclinic, *P*1	*D*_x_ = 1.853 Mg m^−^^3^
Hall symbol: -P 1	Mo *K*α radiation, λ = 0.71073 Å
*a* = 9.6434 (1) Å	Cell parameters from 27797 reflections
*b* = 15.4287 (2) Å	θ = 2.6–40.3°
*c* = 16.4161 (2) Å	µ = 1.39 mm^−^^1^
α = 92.1948 (6)°	*T* = 90 K
β = 98.2331 (7)°	Prism, colourless
γ = 91.3097 (6)°	0.40 × 0.35 × 0.15 mm
*V* = 2414.53 (5) Å^3^	

### Data collection

**Table:**

Nonius KappaCCD diffractometer	30125 independent reflections
Radiation source: sealed tube	24699 reflections with *I* > 2σ(*I*)
Horizonally mounted graphite crystal monochromator	*R*_int_ = 0.026
Detector resolution: 9 pixels mm^-1^	θ_max_ = 40.3°, θ_min_ = 2.6°
CCD rotation images, thick slices scans	*h* = −17→17
Absorption correction: multi-scan (*SCALEPACK*; Otwinowski & Minor, 1997)	*k* = −28→28
*T*_min_ = 0.607, *T*_max_ = 0.819	*l* = −29→29
111692 measured reflections	

### Refinement

**Table:**

Refinement on *F*^2^	Secondary atom site location: difference Fourier map
Least-squares matrix: full	Hydrogen site location: inferred from neighbouring sites
*R*[*F*^2^ > 2σ(*F*^2^)] = 0.032	H-atom parameters constrained
*wR*(*F*^2^) = 0.086	*w* = 1/[σ^2^(*F*_o_^2^) + (0.0362*P*)^2^ + 1.2577*P*] where *P* = (*F*_o_^2^ + 2*F*_c_^2^)/3
*S* = 1.03	(Δ/σ)_max_ = 0.002
30125 reflections	Δρ_max_ = 0.87 e Å^−^^3^
578 parameters	Δρ_min_ = −0.63 e Å^−^^3^
0 restraints	Extinction correction: *SHELXL97* (Sheldrick, 2008), Fc^*^=kFc[1+0.001xFc^2^λ^3^/sin(2θ)]^-1/4^
0 constraints	Extinction coefficient: 0.0013 (2)
Primary atom site location: structure-invariant direct methods	

### Special details

**Table:**

Geometry. All e.s.d.'s (except the e.s.d. in the dihedral angle between two l.s. planes) are estimated using the full covariance matrix. The cell e.s.d.'s are taken into account individually in the estimation of e.s.d.'s in distances, angles and torsion angles; correlations between e.s.d.'s in cell parameters are only used when they are defined by crystal symmetry. An approximate (isotropic) treatment of cell e.s.d.'s is used for estimating e.s.d.'s involving l.s. planes.

### Fractional atomic coordinates and isotropic or equivalent isotropic displacement parameters (Å^2^)

**Table:**

	*x*	*y*	*z*	*U*_iso_*/*U*_eq_	
C1	0.78302 (10)	0.24744 (6)	0.36584 (6)	0.01060 (13)	
H1	0.718	0.2785	0.3984	0.013*	
C2	0.76826 (10)	0.15090 (6)	0.37568 (6)	0.01118 (14)	
C3	0.77164 (12)	0.12163 (7)	0.45581 (6)	0.01484 (16)	
H3	0.7646	0.1622	0.4999	0.018*	
C4	0.78510 (13)	0.03398 (7)	0.47153 (7)	0.01839 (18)	
H4	0.7853	0.0148	0.5258	0.022*	
C5	0.79834 (13)	−0.02536 (7)	0.40736 (7)	0.01770 (18)	
H5	0.8149	−0.0845	0.4183	0.021*	
C6	0.78719 (12)	0.00244 (7)	0.32715 (7)	0.01483 (16)	
H6	0.7938	−0.0386	0.2833	0.018*	
C7	0.76641 (10)	0.08995 (6)	0.30980 (6)	0.01141 (14)	
C8	0.72700 (10)	0.11372 (6)	0.22086 (6)	0.01102 (13)	
H8	0.7957	0.0893	0.1866	0.013*	
C9	0.70962 (10)	0.21274 (6)	0.20593 (6)	0.01055 (13)	
H9	0.7587	0.2264	0.1581	0.013*	
C10	0.76252 (10)	0.27912 (6)	0.27577 (6)	0.01032 (13)	
H10	0.6952	0.3276	0.273	0.012*	
C11	0.91010 (10)	0.31842 (6)	0.26664 (6)	0.01082 (13)	
C12	0.95297 (10)	0.36467 (6)	0.35265 (6)	0.01182 (14)	
C13	0.94026 (10)	0.27907 (6)	0.39788 (6)	0.01102 (13)	
C14	1.03221 (10)	0.22190 (6)	0.35207 (6)	0.01196 (14)	
C15	1.01323 (10)	0.24454 (7)	0.27332 (6)	0.01183 (14)	
C16	0.54883 (10)	0.21886 (6)	0.17658 (6)	0.01145 (14)	
C17	0.52148 (10)	0.13722 (6)	0.11775 (6)	0.01270 (14)	
C18	0.57301 (10)	0.07562 (6)	0.18742 (6)	0.01136 (14)	
C19	0.48189 (10)	0.10455 (6)	0.25116 (6)	0.01187 (14)	
C20	0.47091 (10)	0.19063 (6)	0.24619 (6)	0.01197 (14)	
Cl1	0.83666 (3)	0.444688 (16)	0.378408 (16)	0.01515 (4)	
Cl2	1.12467 (3)	0.411451 (17)	0.367166 (16)	0.01537 (4)	
Cl3	0.98690 (3)	0.288372 (18)	0.504965 (14)	0.01546 (4)	
Cl4	1.12522 (3)	0.138821 (18)	0.394002 (17)	0.01819 (5)	
Cl5	1.07638 (3)	0.195595 (19)	0.192404 (16)	0.01775 (4)	
Cl6	0.91267 (3)	0.383648 (17)	0.181692 (15)	0.01553 (4)	
Cl7	0.62374 (3)	0.132935 (18)	0.036646 (15)	0.01693 (4)	
Cl8	0.34348 (3)	0.119848 (18)	0.074104 (16)	0.01669 (4)	
Cl9	0.56086 (3)	−0.034447 (16)	0.156692 (16)	0.01555 (4)	
Cl10	0.42601 (3)	0.039085 (17)	0.321116 (17)	0.01683 (4)	
Cl11	0.40182 (3)	0.259640 (17)	0.311679 (18)	0.01724 (4)	
Cl12	0.49903 (3)	0.317864 (16)	0.134127 (17)	0.01638 (4)	
C21	0.70185 (10)	0.39226 (6)	0.72349 (6)	0.01099 (13)	
H21	0.7764	0.4224	0.6977	0.013*	
C22	0.72147 (10)	0.41473 (6)	0.81506 (6)	0.01236 (14)	
C23	0.73181 (12)	0.50254 (7)	0.84174 (7)	0.01735 (18)	
H23	0.7441	0.5457	0.8035	0.021*	
C24	0.72439 (14)	0.52707 (8)	0.92324 (8)	0.0220 (2)	
H24	0.7347	0.5865	0.9408	0.026*	
C25	0.70191 (15)	0.46452 (9)	0.97910 (7)	0.0227 (2)	
H25	0.6883	0.4814	1.0336	0.027*	
C26	0.69956 (13)	0.37723 (8)	0.95468 (7)	0.01833 (18)	
H26	0.6872	0.3344	0.9933	0.022*	
C27	0.71524 (11)	0.35144 (7)	0.87372 (6)	0.01303 (15)	
C28	0.74356 (10)	0.25743 (6)	0.85610 (6)	0.01152 (14)	
H28	0.6781	0.2197	0.8822	0.014*	
C29	0.73930 (10)	0.22747 (6)	0.76363 (6)	0.01075 (13)	
H29	0.6754	0.1751	0.7523	0.013*	
C30	0.69489 (10)	0.29315 (6)	0.69746 (6)	0.01106 (13)	
H30	0.7566	0.2851	0.6539	0.013*	
C31	0.54023 (10)	0.27880 (6)	0.65463 (6)	0.01190 (14)	
C32	0.52032 (10)	0.36220 (6)	0.60443 (6)	0.01211 (14)	
C33	0.55125 (10)	0.42229 (6)	0.68299 (6)	0.01044 (13)	
C34	0.44750 (10)	0.38391 (6)	0.73405 (6)	0.01182 (14)	
C35	0.44278 (10)	0.29802 (6)	0.71796 (6)	0.01284 (15)	
C36	0.89301 (10)	0.19842 (6)	0.75984 (6)	0.01145 (14)	
C37	0.92908 (10)	0.15480 (6)	0.84375 (6)	0.01255 (14)	
C38	0.90153 (10)	0.23844 (6)	0.89310 (6)	0.01190 (14)	
C39	0.99247 (10)	0.30344 (7)	0.85577 (6)	0.01231 (14)	
C40	0.98700 (10)	0.27995 (7)	0.77598 (6)	0.01191 (14)	
Cl13	0.64205 (3)	0.377793 (19)	0.534965 (15)	0.01720 (4)	
Cl14	0.34960 (3)	0.370694 (18)	0.549642 (16)	0.01676 (4)	
Cl15	0.53663 (3)	0.532852 (15)	0.665173 (16)	0.01426 (4)	
Cl16	0.37045 (3)	0.441131 (18)	0.804244 (17)	0.01757 (4)	
Cl17	0.35863 (3)	0.221032 (19)	0.76498 (2)	0.02185 (5)	
Cl18	0.50770 (3)	0.180207 (17)	0.597606 (18)	0.01900 (5)	
Cl19	0.81665 (3)	0.066381 (16)	0.857572 (17)	0.01663 (4)	
Cl20	1.10430 (3)	0.120062 (18)	0.864312 (16)	0.01591 (4)	
Cl21	0.93820 (3)	0.232330 (19)	1.000170 (15)	0.01646 (4)	
Cl22	1.06791 (3)	0.393670 (18)	0.906893 (16)	0.01752 (4)	
Cl23	1.05143 (3)	0.336268 (18)	0.701995 (15)	0.01620 (4)	
Cl24	0.91419 (3)	0.134541 (17)	0.672205 (15)	0.01600 (4)	

### Atomic displacement parameters (Å^2^)

**Table:**

	*U*^11^	*U*^22^	*U*^33^	*U*^12^	*U*^13^	*U*^23^
C1	0.0102 (3)	0.0109 (3)	0.0109 (3)	0.0006 (3)	0.0022 (3)	0.0009 (2)
C2	0.0118 (3)	0.0111 (3)	0.0109 (3)	0.0004 (3)	0.0023 (3)	0.0009 (3)
C3	0.0197 (4)	0.0137 (4)	0.0116 (3)	−0.0007 (3)	0.0036 (3)	0.0020 (3)
C4	0.0258 (5)	0.0151 (4)	0.0146 (4)	0.0001 (4)	0.0030 (4)	0.0048 (3)
C5	0.0224 (5)	0.0122 (4)	0.0182 (4)	0.0020 (3)	0.0009 (4)	0.0033 (3)
C6	0.0169 (4)	0.0117 (3)	0.0154 (4)	0.0024 (3)	0.0005 (3)	0.0007 (3)
C7	0.0113 (3)	0.0111 (3)	0.0118 (3)	0.0014 (3)	0.0014 (3)	0.0011 (3)
C8	0.0102 (3)	0.0114 (3)	0.0113 (3)	0.0008 (3)	0.0013 (3)	−0.0006 (3)
C9	0.0093 (3)	0.0116 (3)	0.0107 (3)	−0.0004 (3)	0.0010 (3)	0.0012 (3)
C10	0.0092 (3)	0.0109 (3)	0.0107 (3)	0.0001 (2)	0.0012 (3)	0.0010 (2)
C11	0.0097 (3)	0.0124 (3)	0.0102 (3)	−0.0006 (3)	0.0010 (3)	0.0018 (3)
C12	0.0107 (3)	0.0124 (3)	0.0120 (3)	−0.0009 (3)	0.0008 (3)	0.0006 (3)
C13	0.0104 (3)	0.0132 (3)	0.0094 (3)	−0.0001 (3)	0.0011 (3)	0.0009 (3)
C14	0.0100 (3)	0.0144 (4)	0.0118 (3)	0.0023 (3)	0.0017 (3)	0.0026 (3)
C15	0.0098 (3)	0.0157 (4)	0.0103 (3)	0.0012 (3)	0.0023 (3)	0.0009 (3)
C16	0.0106 (3)	0.0108 (3)	0.0125 (3)	0.0003 (3)	−0.0001 (3)	0.0011 (3)
C17	0.0118 (4)	0.0140 (4)	0.0117 (3)	−0.0009 (3)	−0.0003 (3)	0.0002 (3)
C18	0.0111 (3)	0.0106 (3)	0.0123 (3)	0.0000 (3)	0.0016 (3)	−0.0009 (3)
C19	0.0106 (3)	0.0115 (3)	0.0139 (4)	−0.0002 (3)	0.0031 (3)	0.0004 (3)
C20	0.0099 (3)	0.0114 (3)	0.0147 (4)	0.0009 (3)	0.0023 (3)	−0.0003 (3)
Cl1	0.01452 (9)	0.01185 (8)	0.01893 (10)	0.00055 (7)	0.00233 (8)	−0.00109 (7)
Cl2	0.01118 (9)	0.01763 (10)	0.01655 (9)	−0.00436 (7)	−0.00006 (7)	0.00173 (7)
Cl3	0.01539 (10)	0.02149 (10)	0.00901 (8)	−0.00196 (8)	0.00049 (7)	0.00051 (7)
Cl4	0.01609 (10)	0.02054 (11)	0.01883 (10)	0.00766 (8)	0.00282 (8)	0.00687 (8)
Cl5	0.01531 (10)	0.02630 (12)	0.01232 (9)	0.00480 (8)	0.00425 (7)	−0.00134 (8)
Cl6	0.01548 (10)	0.01764 (10)	0.01337 (9)	−0.00241 (7)	0.00075 (7)	0.00634 (7)
Cl7	0.01755 (10)	0.02238 (11)	0.01071 (9)	−0.00275 (8)	0.00244 (7)	−0.00091 (7)
Cl8	0.01222 (9)	0.01876 (10)	0.01734 (10)	−0.00185 (7)	−0.00323 (7)	−0.00030 (8)
Cl9	0.01529 (10)	0.01106 (8)	0.01946 (10)	0.00016 (7)	0.00067 (8)	−0.00321 (7)
Cl10	0.01749 (10)	0.01530 (9)	0.01925 (10)	−0.00123 (8)	0.00744 (8)	0.00352 (8)
Cl11	0.01515 (10)	0.01373 (9)	0.02399 (11)	0.00053 (7)	0.00797 (8)	−0.00388 (8)
Cl12	0.01552 (10)	0.01303 (9)	0.01943 (10)	0.00099 (7)	−0.00262 (8)	0.00466 (7)
C21	0.0096 (3)	0.0118 (3)	0.0114 (3)	−0.0004 (3)	0.0008 (3)	0.0013 (3)
C22	0.0120 (4)	0.0123 (3)	0.0120 (3)	0.0003 (3)	−0.0007 (3)	−0.0008 (3)
C23	0.0193 (4)	0.0132 (4)	0.0171 (4)	0.0014 (3)	−0.0051 (3)	−0.0015 (3)
C24	0.0272 (6)	0.0168 (4)	0.0187 (5)	0.0065 (4)	−0.0075 (4)	−0.0061 (3)
C25	0.0280 (6)	0.0235 (5)	0.0151 (4)	0.0084 (4)	−0.0015 (4)	−0.0061 (4)
C26	0.0215 (5)	0.0212 (5)	0.0122 (4)	0.0045 (4)	0.0021 (3)	−0.0012 (3)
C27	0.0133 (4)	0.0143 (4)	0.0112 (3)	0.0016 (3)	0.0010 (3)	−0.0004 (3)
C28	0.0106 (3)	0.0135 (3)	0.0109 (3)	0.0004 (3)	0.0023 (3)	0.0022 (3)
C29	0.0093 (3)	0.0118 (3)	0.0110 (3)	0.0011 (3)	0.0008 (3)	0.0008 (3)
C30	0.0099 (3)	0.0120 (3)	0.0110 (3)	0.0008 (3)	0.0007 (3)	−0.0001 (3)
C31	0.0107 (3)	0.0109 (3)	0.0132 (3)	0.0005 (3)	−0.0010 (3)	−0.0005 (3)
C32	0.0116 (3)	0.0140 (3)	0.0103 (3)	0.0013 (3)	0.0001 (3)	0.0006 (3)
C33	0.0093 (3)	0.0107 (3)	0.0114 (3)	0.0002 (2)	0.0014 (3)	0.0012 (3)
C34	0.0098 (3)	0.0135 (3)	0.0124 (3)	0.0001 (3)	0.0025 (3)	0.0012 (3)
C35	0.0098 (3)	0.0123 (3)	0.0167 (4)	−0.0009 (3)	0.0023 (3)	0.0032 (3)
C36	0.0101 (3)	0.0137 (3)	0.0102 (3)	0.0017 (3)	0.0005 (3)	0.0002 (3)
C37	0.0104 (3)	0.0143 (4)	0.0129 (3)	0.0014 (3)	0.0010 (3)	0.0028 (3)
C38	0.0113 (3)	0.0152 (4)	0.0092 (3)	0.0000 (3)	0.0013 (3)	0.0021 (3)
C39	0.0108 (3)	0.0152 (4)	0.0106 (3)	−0.0017 (3)	0.0008 (3)	0.0013 (3)
C40	0.0103 (3)	0.0160 (4)	0.0096 (3)	−0.0006 (3)	0.0015 (3)	0.0023 (3)
Cl13	0.01770 (10)	0.02354 (11)	0.01141 (9)	0.00470 (8)	0.00434 (7)	0.00335 (8)
Cl14	0.01352 (9)	0.01905 (10)	0.01587 (10)	0.00283 (8)	−0.00431 (7)	0.00008 (8)
Cl15	0.01306 (9)	0.01089 (8)	0.01855 (10)	0.00068 (7)	0.00050 (7)	0.00358 (7)
Cl16	0.01555 (10)	0.02152 (11)	0.01677 (10)	0.00244 (8)	0.00659 (8)	−0.00163 (8)
Cl17	0.01655 (11)	0.01744 (11)	0.03321 (14)	−0.00172 (8)	0.00695 (10)	0.01084 (10)
Cl18	0.01851 (11)	0.01363 (9)	0.02210 (11)	0.00153 (8)	−0.00507 (9)	−0.00519 (8)
Cl19	0.01466 (10)	0.01355 (9)	0.02162 (11)	−0.00028 (7)	0.00144 (8)	0.00481 (8)
Cl20	0.01137 (9)	0.02036 (10)	0.01609 (9)	0.00441 (7)	0.00072 (7)	0.00441 (8)
Cl21	0.01617 (10)	0.02432 (11)	0.00905 (8)	0.00128 (8)	0.00142 (7)	0.00381 (7)
Cl22	0.01692 (10)	0.01942 (10)	0.01496 (9)	−0.00570 (8)	−0.00048 (8)	−0.00125 (8)
Cl23	0.01377 (9)	0.02338 (11)	0.01183 (9)	−0.00289 (8)	0.00262 (7)	0.00475 (8)
Cl24	0.01656 (10)	0.01830 (10)	0.01279 (9)	0.00548 (8)	0.00096 (7)	−0.00253 (7)

### Geometric parameters (Å, º)

**Table:**

C1—C2	1.5099 (13)	C21—C22	1.5136 (14)
C1—C10	1.5623 (13)	C21—C30	1.5694 (13)
C1—C13	1.5913 (13)	C21—C33	1.5949 (13)
C1—H1	1	C21—H21	1
C2—C3	1.4033 (14)	C22—C27	1.4037 (14)
C2—C7	1.4036 (14)	C22—C23	1.4046 (14)
C3—C4	1.3919 (15)	C23—C24	1.3885 (17)
C3—H3	0.95	C23—H23	0.95
C4—C5	1.3908 (17)	C24—C25	1.391 (2)
C4—H4	0.95	C24—H24	0.95
C5—C6	1.3907 (15)	C25—C26	1.3891 (17)
C5—H5	0.95	C25—H25	0.95
C6—C7	1.4035 (14)	C26—C27	1.4026 (15)
C6—H6	0.95	C26—H26	0.95
C7—C8	1.5163 (13)	C27—C28	1.5070 (14)
C8—C9	1.5655 (13)	C28—C29	1.5643 (13)
C8—C18	1.5980 (13)	C28—C38	1.5951 (14)
C8—H8	1	C28—H28	1
C9—C10	1.5305 (13)	C29—C30	1.5366 (13)
C9—C16	1.5625 (13)	C29—C36	1.5673 (13)
C9—H9	1	C29—H29	1
C10—C11	1.5633 (13)	C30—C31	1.5622 (13)
C10—H10	1	C30—H30	1
C11—C15	1.5265 (14)	C31—C35	1.5233 (15)
C11—C12	1.5543 (13)	C31—C32	1.5554 (14)
C11—Cl6	1.7535 (10)	C31—Cl18	1.7531 (10)
C12—C13	1.5506 (14)	C32—C33	1.5484 (13)
C12—Cl1	1.7664 (10)	C32—Cl13	1.7685 (10)
C12—Cl2	1.7716 (10)	C32—Cl14	1.7695 (10)
C13—C14	1.5185 (14)	C33—C34	1.5183 (13)
C13—Cl3	1.7505 (9)	C33—Cl15	1.7472 (9)
C14—C15	1.3403 (13)	C34—C35	1.3398 (14)
C14—Cl4	1.6926 (10)	C34—Cl16	1.6902 (10)
C15—Cl5	1.6976 (10)	C35—Cl17	1.6943 (10)
C16—C20	1.5262 (14)	C36—C40	1.5256 (14)
C16—C17	1.5517 (14)	C36—C37	1.5544 (13)
C16—Cl12	1.7518 (10)	C36—Cl24	1.7514 (10)
C17—C18	1.5493 (14)	C37—C38	1.5459 (14)
C17—Cl7	1.7668 (11)	C37—Cl19	1.7648 (10)
C17—Cl8	1.7730 (10)	C37—Cl20	1.7744 (10)
C18—C19	1.5204 (14)	C38—C39	1.5202 (14)
C18—Cl9	1.7495 (10)	C38—Cl21	1.7492 (10)
C19—C20	1.3395 (14)	C39—C40	1.3390 (14)
C19—Cl10	1.6943 (10)	C39—Cl22	1.6915 (10)
C20—Cl11	1.6980 (10)	C40—Cl23	1.6995 (10)
			
C2—C1—C10	116.62 (8)	C22—C21—C30	116.46 (8)
C2—C1—C13	109.50 (8)	C22—C21—C33	109.13 (8)
C10—C1—C13	101.60 (7)	C30—C21—C33	101.61 (7)
C2—C1—H1	109.6	C22—C21—H21	109.8
C10—C1—H1	109.6	C30—C21—H21	109.8
C13—C1—H1	109.6	C33—C21—H21	109.8
C3—C2—C7	119.20 (9)	C27—C22—C23	118.69 (9)
C3—C2—C1	117.67 (8)	C27—C22—C21	122.21 (9)
C7—C2—C1	122.78 (8)	C23—C22—C21	118.77 (9)
C4—C3—C2	120.93 (10)	C24—C23—C22	120.94 (11)
C4—C3—H3	119.5	C24—C23—H23	119.5
C2—C3—H3	119.5	C22—C23—H23	119.5
C5—C4—C3	119.68 (10)	C23—C24—C25	119.96 (11)
C5—C4—H4	120.2	C23—C24—H24	120
C3—C4—H4	120.2	C25—C24—H24	120
C6—C5—C4	119.63 (10)	C26—C25—C24	119.55 (11)
C6—C5—H5	120.2	C26—C25—H25	120.2
C4—C5—H5	120.2	C24—C25—H25	120.2
C5—C6—C7	121.21 (10)	C25—C26—C27	120.86 (11)
C5—C6—H6	119.4	C25—C26—H26	119.6
C7—C6—H6	119.4	C27—C26—H26	119.6
C6—C7—C2	118.77 (9)	C26—C27—C22	119.35 (10)
C6—C7—C8	118.72 (9)	C26—C27—C28	117.94 (9)
C2—C7—C8	122.16 (8)	C22—C27—C28	122.23 (9)
C7—C8—C9	115.90 (8)	C27—C28—C29	116.94 (8)
C7—C8—C18	109.08 (8)	C27—C28—C38	109.12 (8)
C9—C8—C18	101.61 (7)	C29—C28—C38	101.68 (7)
C7—C8—H8	110	C27—C28—H28	109.6
C9—C8—H8	110	C29—C28—H28	109.6
C18—C8—H8	110	C38—C28—H28	109.6
C10—C9—C16	112.28 (8)	C30—C29—C28	118.17 (8)
C10—C9—C8	119.29 (8)	C30—C29—C36	110.65 (8)
C16—C9—C8	102.75 (7)	C28—C29—C36	102.98 (7)
C10—C9—H9	107.3	C30—C29—H29	108.2
C16—C9—H9	107.3	C28—C29—H29	108.2
C8—C9—H9	107.3	C36—C29—H29	108.2
C9—C10—C1	117.92 (8)	C29—C30—C31	113.40 (8)
C9—C10—C11	111.87 (8)	C29—C30—C21	118.58 (8)
C1—C10—C11	103.20 (7)	C31—C30—C21	102.81 (7)
C9—C10—H10	107.8	C29—C30—H30	107.1
C1—C10—H10	107.8	C31—C30—H30	107.1
C11—C10—H10	107.8	C21—C30—H30	107.1
C15—C11—C12	99.75 (7)	C35—C31—C32	99.48 (8)
C15—C11—C10	107.59 (8)	C35—C31—C30	108.49 (8)
C12—C11—C10	101.23 (7)	C32—C31—C30	100.82 (7)
C15—C11—Cl6	115.79 (7)	C35—C31—Cl18	115.94 (7)
C12—C11—Cl6	115.87 (7)	C32—C31—Cl18	115.82 (7)
C10—C11—Cl6	114.67 (6)	C30—C31—Cl18	114.34 (7)
C13—C12—C11	92.42 (7)	C33—C32—C31	92.48 (7)
C13—C12—Cl1	113.60 (7)	C33—C32—Cl13	112.85 (7)
C11—C12—Cl1	114.64 (7)	C31—C32—Cl13	114.98 (7)
C13—C12—Cl2	113.77 (7)	C33—C32—Cl14	114.30 (7)
C11—C12—Cl2	113.72 (7)	C31—C32—Cl14	113.66 (7)
Cl1—C12—Cl2	108.22 (5)	Cl13—C32—Cl14	108.13 (5)
C14—C13—C12	99.82 (8)	C34—C33—C32	99.98 (7)
C14—C13—C1	106.03 (7)	C34—C33—C21	105.85 (7)
C12—C13—C1	102.45 (7)	C32—C33—C21	102.54 (7)
C14—C13—Cl3	115.75 (7)	C34—C33—Cl15	116.02 (7)
C12—C13—Cl3	114.89 (7)	C32—C33—Cl15	114.41 (7)
C1—C13—Cl3	115.87 (7)	C21—C33—Cl15	116.01 (6)
C15—C14—C13	107.20 (8)	C35—C34—C33	106.63 (8)
C15—C14—Cl4	128.20 (8)	C35—C34—Cl16	128.68 (8)
C13—C14—Cl4	124.28 (7)	C33—C34—Cl16	124.37 (7)
C14—C15—C11	107.22 (8)	C34—C35—C31	107.86 (8)
C14—C15—Cl5	127.72 (8)	C34—C35—Cl17	127.52 (8)
C11—C15—Cl5	124.79 (7)	C31—C35—Cl17	124.34 (7)
C20—C16—C17	99.79 (8)	C40—C36—C37	100.05 (7)
C20—C16—C9	108.31 (8)	C40—C36—C29	106.76 (8)
C17—C16—C9	100.91 (8)	C37—C36—C29	101.62 (8)
C20—C16—Cl12	115.58 (7)	C40—C36—Cl24	115.80 (7)
C17—C16—Cl12	116.17 (7)	C37—C36—Cl24	115.92 (7)
C9—C16—Cl12	114.22 (7)	C29—C36—Cl24	114.76 (6)
C18—C17—C16	92.43 (7)	C38—C37—C36	92.52 (7)
C18—C17—Cl7	112.78 (7)	C38—C37—Cl19	113.76 (7)
C16—C17—Cl7	114.95 (7)	C36—C37—Cl19	114.29 (7)
C18—C17—Cl8	114.39 (7)	C38—C37—Cl20	113.72 (7)
C16—C17—Cl8	114.08 (7)	C36—C37—Cl20	114.20 (7)
Cl7—C17—Cl8	107.83 (5)	Cl19—C37—Cl20	107.95 (5)
C19—C18—C17	99.77 (8)	C39—C38—C37	100.27 (8)
C19—C18—C8	105.71 (7)	C39—C38—C28	105.99 (8)
C17—C18—C8	102.71 (7)	C37—C38—C28	101.98 (7)
C19—C18—Cl9	116.46 (7)	C39—C38—Cl21	115.08 (7)
C17—C18—Cl9	114.10 (7)	C37—C38—Cl21	114.92 (7)
C8—C18—Cl9	116.01 (7)	C28—C38—Cl21	116.56 (7)
C20—C19—C18	106.61 (8)	C40—C39—C38	107.24 (8)
C20—C19—Cl10	128.56 (8)	C40—C39—Cl22	128.51 (8)
C18—C19—Cl10	124.41 (7)	C38—C39—Cl22	123.90 (7)
C19—C20—C16	107.69 (8)	C39—C40—C36	107.17 (8)
C19—C20—Cl11	127.26 (8)	C39—C40—Cl23	127.44 (8)
C16—C20—Cl11	124.64 (7)	C36—C40—Cl23	124.86 (7)
			
C10—C1—C2—C3	−173.50 (9)	C30—C21—C22—C27	−8.24 (14)
C13—C1—C2—C3	71.92 (11)	C33—C21—C22—C27	106.00 (10)
C10—C1—C2—C7	13.41 (13)	C30—C21—C22—C23	178.47 (9)
C13—C1—C2—C7	−101.18 (10)	C33—C21—C22—C23	−67.29 (12)
C7—C2—C3—C4	5.48 (16)	C27—C22—C23—C24	−5.27 (16)
C1—C2—C3—C4	−167.88 (10)	C21—C22—C23—C24	168.26 (10)
C2—C3—C4—C5	1.35 (18)	C22—C23—C24—C25	−2.09 (19)
C3—C4—C5—C6	−4.92 (18)	C23—C24—C25—C26	5.7 (2)
C4—C5—C6—C7	1.69 (18)	C24—C25—C26—C27	−1.98 (19)
C5—C6—C7—C2	5.11 (16)	C25—C26—C27—C22	−5.41 (17)
C5—C6—C7—C8	−168.25 (10)	C25—C26—C27—C28	166.77 (11)
C3—C2—C7—C6	−8.57 (15)	C23—C22—C27—C26	8.92 (15)
C1—C2—C7—C6	164.42 (9)	C21—C22—C27—C26	−164.38 (10)
C3—C2—C7—C8	164.54 (9)	C23—C22—C27—C28	−162.92 (10)
C1—C2—C7—C8	−22.46 (15)	C21—C22—C27—C28	23.78 (15)
C6—C7—C8—C9	−178.35 (9)	C26—C27—C28—C29	171.49 (9)
C2—C7—C8—C9	8.53 (13)	C22—C27—C28—C29	−16.56 (14)
C6—C7—C8—C18	67.77 (11)	C26—C27—C28—C38	−73.90 (12)
C2—C7—C8—C18	−105.35 (10)	C22—C27—C28—C38	98.05 (11)
C7—C8—C9—C10	13.07 (12)	C27—C28—C29—C30	−5.02 (12)
C18—C8—C9—C10	131.16 (8)	C38—C28—C29—C30	−123.72 (9)
C7—C8—C9—C16	−111.87 (9)	C27—C28—C29—C36	117.26 (9)
C18—C8—C9—C16	6.21 (9)	C38—C28—C29—C36	−1.44 (9)
C16—C9—C10—C1	99.28 (9)	C28—C29—C30—C31	−101.76 (10)
C8—C9—C10—C1	−20.94 (12)	C36—C29—C30—C31	139.94 (8)
C16—C9—C10—C11	−141.30 (8)	C28—C29—C30—C21	18.97 (12)
C8—C9—C10—C11	98.47 (10)	C36—C29—C30—C21	−99.33 (10)
C2—C1—C10—C9	8.32 (12)	C22—C21—C30—C29	−12.98 (12)
C13—C1—C10—C9	127.27 (8)	C33—C21—C30—C29	−131.41 (8)
C2—C1—C10—C11	−115.55 (9)	C22—C21—C30—C31	113.02 (9)
C13—C1—C10—C11	3.40 (9)	C33—C21—C30—C31	−5.41 (9)
C9—C10—C11—C15	−63.17 (9)	C29—C30—C31—C35	66.67 (10)
C1—C10—C11—C15	64.59 (9)	C21—C30—C31—C35	−62.61 (9)
C9—C10—C11—C12	−167.29 (7)	C29—C30—C31—C32	170.59 (8)
C1—C10—C11—C12	−39.54 (9)	C21—C30—C31—C32	41.31 (9)
C9—C10—C11—Cl6	67.21 (9)	C29—C30—C31—Cl18	−64.44 (10)
C1—C10—C11—Cl6	−165.03 (6)	C21—C30—C31—Cl18	166.28 (7)
C15—C11—C12—C13	−51.76 (8)	C35—C31—C32—C33	51.48 (8)
C10—C11—C12—C13	58.53 (8)	C30—C31—C32—C33	−59.58 (8)
Cl6—C11—C12—C13	−176.78 (7)	Cl18—C31—C32—C33	176.46 (7)
C15—C11—C12—Cl1	−169.16 (6)	C35—C31—C32—Cl13	168.16 (6)
C10—C11—C12—Cl1	−58.87 (8)	C30—C31—C32—Cl13	57.11 (9)
Cl6—C11—C12—Cl1	65.82 (9)	Cl18—C31—C32—Cl13	−66.86 (9)
C15—C11—C12—Cl2	65.58 (8)	C35—C31—C32—Cl14	−66.47 (8)
C10—C11—C12—Cl2	175.87 (6)	C30—C31—C32—Cl14	−177.53 (6)
Cl6—C11—C12—Cl2	−59.44 (9)	Cl18—C31—C32—Cl14	58.51 (9)
C11—C12—C13—C14	52.50 (8)	C31—C32—C33—C34	−52.74 (8)
Cl1—C12—C13—C14	170.78 (6)	Cl13—C32—C33—C34	−171.23 (6)
Cl2—C12—C13—C14	−64.80 (8)	Cl14—C32—C33—C34	64.66 (8)
C11—C12—C13—C1	−56.48 (8)	C31—C32—C33—C21	56.12 (8)
Cl1—C12—C13—C1	61.80 (8)	Cl13—C32—C33—C21	−62.37 (8)
Cl2—C12—C13—C1	−173.78 (6)	Cl14—C32—C33—C21	173.52 (6)
C11—C12—C13—Cl3	177.01 (7)	C31—C32—C33—Cl15	−177.42 (6)
Cl1—C12—C13—Cl3	−64.72 (8)	Cl13—C32—C33—Cl15	64.09 (8)
Cl2—C12—C13—Cl3	59.71 (9)	Cl14—C32—C33—Cl15	−60.01 (9)
C2—C1—C13—C14	53.64 (9)	C22—C21—C33—C34	−51.60 (10)
C10—C1—C13—C14	−70.27 (8)	C30—C21—C33—C34	71.95 (9)
C2—C1—C13—C12	157.83 (8)	C22—C21—C33—C32	−155.94 (8)
C10—C1—C13—C12	33.93 (9)	C30—C21—C33—C32	−32.39 (9)
C2—C1—C13—Cl3	−76.28 (9)	C22—C21—C33—Cl15	78.63 (9)
C10—C1—C13—Cl3	159.81 (6)	C30—C21—C33—Cl15	−157.82 (7)
C12—C13—C14—C15	−35.79 (10)	C32—C33—C34—C35	36.13 (10)
C1—C13—C14—C15	70.31 (10)	C21—C33—C34—C35	−70.08 (10)
Cl3—C13—C14—C15	−159.70 (7)	Cl15—C33—C34—C35	159.69 (7)
C12—C13—C14—Cl4	150.16 (8)	C32—C33—C34—Cl16	−149.88 (7)
C1—C13—C14—Cl4	−103.74 (9)	C21—C33—C34—Cl16	103.91 (9)
Cl3—C13—C14—Cl4	26.25 (11)	Cl15—C33—C34—Cl16	−26.32 (11)
C13—C14—C15—C11	1.03 (11)	C33—C34—C35—C31	−1.40 (11)
Cl4—C14—C15—C11	174.77 (8)	Cl16—C34—C35—C31	−175.04 (8)
C13—C14—C15—Cl5	−173.21 (8)	C33—C34—C35—Cl17	172.77 (8)
Cl4—C14—C15—Cl5	0.53 (15)	Cl16—C34—C35—Cl17	−0.88 (15)
C12—C11—C15—C14	33.98 (10)	C32—C31—C35—C34	−33.58 (10)
C10—C11—C15—C14	−71.19 (10)	C30—C31—C35—C34	71.28 (10)
Cl6—C11—C15—C14	159.06 (7)	Cl18—C31—C35—C34	−158.48 (7)
C12—C11—C15—Cl5	−151.57 (8)	C32—C31—C35—Cl17	152.03 (7)
C10—C11—C15—Cl5	103.26 (9)	C30—C31—C35—Cl17	−103.11 (9)
Cl6—C11—C15—Cl5	−26.49 (11)	Cl18—C31—C35—Cl17	27.12 (11)
C10—C9—C16—C20	−67.18 (10)	C30—C29—C36—C40	60.71 (9)
C8—C9—C16—C20	62.24 (9)	C28—C29—C36—C40	−66.49 (9)
C10—C9—C16—C17	−171.43 (8)	C30—C29—C36—C37	165.07 (8)
C8—C9—C16—C17	−42.02 (9)	C28—C29—C36—C37	37.87 (9)
C10—C9—C16—Cl12	63.17 (9)	C30—C29—C36—Cl24	−69.04 (9)
C8—C9—C16—Cl12	−167.41 (6)	C28—C29—C36—Cl24	163.76 (6)
C20—C16—C17—C18	−51.30 (8)	C40—C36—C37—C38	51.35 (8)
C9—C16—C17—C18	59.68 (8)	C29—C36—C37—C38	−58.25 (8)
Cl12—C16—C17—C18	−176.26 (7)	Cl24—C36—C37—C38	176.64 (7)
C20—C16—C17—Cl7	−167.86 (7)	C40—C36—C37—Cl19	168.89 (7)
C9—C16—C17—Cl7	−56.89 (9)	C29—C36—C37—Cl19	59.29 (9)
Cl12—C16—C17—Cl7	67.18 (9)	Cl24—C36—C37—Cl19	−65.83 (9)
C20—C16—C17—Cl8	66.84 (8)	C40—C36—C37—Cl20	−66.11 (9)
C9—C16—C17—Cl8	177.82 (6)	C29—C36—C37—Cl20	−175.71 (6)
Cl12—C16—C17—Cl8	−58.12 (9)	Cl24—C36—C37—Cl20	59.17 (9)
C16—C17—C18—C19	53.07 (8)	C36—C37—C38—C39	−51.74 (8)
Cl7—C17—C18—C19	171.48 (6)	Cl19—C37—C38—C39	−169.73 (6)
Cl8—C17—C18—C19	−64.80 (8)	Cl20—C37—C38—C39	66.13 (8)
C16—C17—C18—C8	−55.62 (8)	C36—C37—C38—C28	57.20 (8)
Cl7—C17—C18—C8	62.79 (8)	Cl19—C37—C38—C28	−60.78 (8)
Cl8—C17—C18—C8	−173.50 (6)	Cl20—C37—C38—C28	175.07 (6)
C16—C17—C18—Cl9	177.98 (7)	C36—C37—C38—Cl21	−175.75 (7)
Cl7—C17—C18—Cl9	−63.61 (8)	Cl19—C37—C38—Cl21	66.27 (8)
Cl8—C17—C18—Cl9	60.10 (9)	Cl20—C37—C38—Cl21	−57.87 (9)
C7—C8—C18—C19	50.31 (10)	C27—C28—C38—C39	−55.30 (10)
C9—C8—C18—C19	−72.58 (9)	C29—C28—C38—C39	68.84 (9)
C7—C8—C18—C17	154.44 (8)	C27—C28—C38—C37	−159.80 (8)
C9—C8—C18—C17	31.56 (9)	C29—C28—C38—C37	−35.66 (9)
C7—C8—C18—Cl9	−80.40 (9)	C27—C28—C38—Cl21	74.21 (9)
C9—C8—C18—Cl9	156.71 (7)	C29—C28—C38—Cl21	−161.65 (7)
C17—C18—C19—C20	−37.05 (10)	C37—C38—C39—C40	35.04 (10)
C8—C18—C19—C20	69.24 (10)	C28—C38—C39—C40	−70.71 (10)
Cl9—C18—C19—C20	−160.31 (7)	Cl21—C38—C39—C40	158.93 (8)
C17—C18—C19—Cl10	149.88 (7)	C37—C38—C39—Cl22	−151.30 (8)
C8—C18—C19—Cl10	−103.83 (9)	C28—C38—C39—Cl22	102.96 (9)
Cl9—C18—C19—Cl10	26.62 (11)	Cl21—C38—C39—Cl22	−27.41 (11)
C18—C19—C20—C16	2.56 (10)	C38—C39—C40—C36	−0.47 (11)
Cl10—C19—C20—C16	175.25 (8)	Cl22—C39—C40—C36	−173.75 (8)
C18—C19—C20—Cl11	−170.33 (7)	C38—C39—C40—Cl23	171.46 (8)
Cl10—C19—C20—Cl11	2.35 (15)	Cl22—C39—C40—Cl23	−1.82 (16)
C17—C16—C20—C19	32.74 (10)	C37—C36—C40—C39	−34.02 (10)
C9—C16—C20—C19	−72.30 (10)	C29—C36—C40—C39	71.46 (10)
Cl12—C16—C20—C19	158.11 (7)	Cl24—C36—C40—C39	−159.39 (7)
C17—C16—C20—Cl11	−154.13 (7)	C37—C36—C40—Cl23	153.79 (8)
C9—C16—C20—Cl11	100.83 (9)	C29—C36—C40—Cl23	−100.73 (9)
Cl12—C16—C20—Cl11	−28.77 (11)	Cl24—C36—C40—Cl23	28.42 (11)
